# Diffusion and Gas Flow Dynamics in Partially Saturated Smectites

**DOI:** 10.1021/acs.jpcc.3c02264

**Published:** 2023-07-17

**Authors:** Jerry P. Owusu, Konstantinos Karalis, Nikolaos I. Prasianakis, Sergey V. Churakov

**Affiliations:** † Laboratory for Waste Management, Paul Scherrer Institute, 5232 Villigen-PSI, Switzerland; ‡ Institute of Geological Sciences, University of Bern, 3012 Bern, Switzerland

## Abstract

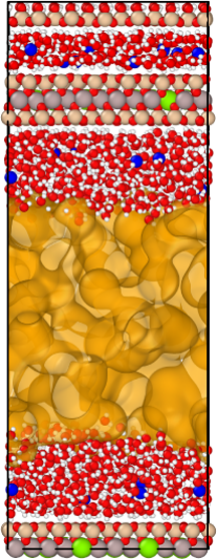

Clays and clay rocks are considered good natural and engineered barriers for deep geological disposal of nuclear waste worldwide. Metal corrosion and organic waste degradation in underground repositories generate significant amounts of gas that should be able to migrate through the multibarrier system to avoid potential pressure buildup, which could be compromising the integrity of the barriers and host rocks. The gas is expected to accumulate in larger pores and eventually form an interconnected network. Under such conditions, the migration of gas molecules takes place both in pore water films and gas-filled macropores. Therefore, mass fluxes depend on the distribution of gas molecules between the water-rich and gas-rich phases and their mobility in both compartments. Classical molecular dynamics (MD) simulations were employed to investigate the mobilities of He, H_2_, CO_2_, Ar, and CH_4_ in a Na-montmorillonite mesopore as a function of the degree of saturation, as well as evaluate the hydrodynamic behavior of the pore fluid in partially saturated clays. The diffusivity of the gas molecules was determined by observing the asymptotic behavior of the mean square displacement in the gas-rich phase and at the gas–water interface. The partition coefficient and Gibbs free energy were analyzed to investigate the transfer of gas molecules between the gas-rich and water-rich phases by observing the molecular trajectories as they cross the vapor–liquid interface. The results revealed that the diffusion coefficient in the gas phase increased with increasing gas-filled pore width and converged asymptotically toward the diffusion coefficient in the bulk state. It could be shown that the diffusion coefficient of gas molecules dissolved in the water films remained constant as long as the interacting water surface was in the bulk-liquid-like phase. This behavior changes in very thin water films. It was observed that the partitioning coefficient of gas molecules at the solid–liquid interface is nearly the same as that in the bulk-liquid-like phase. Partitioning is observed to be strongly dependent on the temperature and gas molecular weights. In the second part of the study, nonequilibrium molecular dynamics (NEMD) simulations were performed to investigate the mobility of gases in pressure-driven decoupled gas-phase dynamics (DGPD) and coupled gas and water phase dynamics (CGWPD) in a partially saturated Na-montmorillonite slit mesopore. The dynamic viscosity of the gas phase was calculated from NEMD simulations and indicated that the viscosity of the gas phase was almost the same in both methods (DGPD and CGWPD). The average slip length for gas molecules at the gas–water interface was also calculated, revealing that the slip-free boundary condition assumed in continuum models is generally invalid for microfluidics and that a slip boundary condition exists at the microscale for specific surface interactions. Finally, a Bosanquet-type equation was developed to predict the diffusion coefficient and dynamic viscosity of gas as a function of the average pore width, gas mean-free path, geometric factor, and thickness of the adsorbed water film.

## Introduction

In several countries, clay rocks are considered potential host rocks for the geological disposal of radioactive waste. Clay materials are also considered as backfill materials and as buffer materials due to their low permeability and adsorption capacity of their charged surfaces.^1−6^ Gases generated in the repository due to corrosion and biodegradation processes should be able to migrate through the multibarrier system so as not to compromise the integrity of the barrier.^7−9^ Therefore, understanding the mechanism of gas transport from such a system is necessary to ensure the safety of the repository.

Advective transport in low-permeability clay rock is negligible under natural hydraulic conditions in fully saturated rocks.^3,10,11^ Therefore, molecular diffusion is expected to be the predominant gas transport mechanism through the multibarrier system. The low-permeability clay materials are expected to undergo desaturation and resaturation phases caused by repository ventilation, thermally induced effects of the repository, or gas production.^12,13^ Unconnected pores thus facilitate the diffusion mechanism at different degrees of saturation.^14,15^

Several studies addressed the diffusion of water and tracers in clays in laboratory experiments.^16−23^ Very challenging gas transport experiments have also been conducted in the laboratory and in the field.^24−31^ Gas diffusion experiments were supported and proved by numerical and molecular multiscale simulations to gain a deeper understanding of the diffusion mechanism under variable conditions.^32−41^ Most of these studies were investigated under the two limiting conditions: (a) fully saturated and (b) fully dry clay conditions. In a typical repository, gas transport would take place under saturated and partially saturated conditions. It is therefore necessary to understand the mechanism of gas migration in clays under partially saturated conditions. Furthermore, the viscosity of gas in a macropore is expected to depend on the degree of saturation and the properties of gas molecules. Gas diffusivity under saturated conditions was addressed in our earlier study.^42^ Here, the earlier simulation approach is extended to partially saturated conditions.

Due to the hydrophilic nature of smectite clays in particular, a water film is adsorbed on the outer surface of the clay particles during the desaturation of the mesopores. The thickness of the water film depends on the water potential, which is related to the relative humidity of the vapor phase.^15^ Studies have shown that this water film, also called electrical double layer (EDL), is the main transport pathway for ions.^14,43^

In an unsaturated clay medium, the diffusion of gas in a pore can undergo two main diffusion regimes: (a) diffusion in the gas-rich phase and (b) diffusion in the liquid-rich phase. Diffusion of He, CH_4_, Ar, CO_2_, and N_2_ was measured in saturated clays using field experiments and laboratory studies.^24−26,30,31^ It was shown that the diffusion of gases in saturated systems depends on the size of the gas molecules and the geometric factor.^24,31^ Gadikota et al.^39^ performed molecular dynamics (MD) simulations of the diffusion and distribution of noble gases in the interlayer of saturated Na-montmorillonite. The results confirmed the experimental observations that diffusion in clays is slower than in bulk water and that the diffusion and partition coefficients of gases depend on the type and size of the diffusing gas molecule. The partitioning of gases is crucial for understanding the retardation and retention of gases during transport and its influence on the rate and extent of gas movement.^44^

In general, the rate of diffusion is significantly higher in the gas phase compared to the liquid phase. This can be observed from the self-diffusion coefficient of helium (He) at standard conditions, which is 7.1 × 10^–5^ m^2^/s, whereas the diffusion coefficient in water is 7.2 × 10^–9^ m^2^/s.^45,46^ Moreover, when it comes to diffusion in clays, partially saturated clays are expected to exhibit higher diffusion rates than fully saturated ones. A study by Mohammed et al.^47^ demonstrated that the diffusion coefficients of CO_2_ and CH_4_ confined in partially saturated calcite pores were respectively 1 and 2 orders of magnitude higher compared to the diffusion rates in saturated calcite pores. A number of studies have been carried out to investigate gas diffusion at partially saturated conditions, ranging from very loose soil material to highly compacted clay material.^48−53^ Recently, Wesenauer et al.^49^ experimentally investigated the gas-phase diffusion and permeability of O_2_, CO_2_, and N_2_ at different pretreatment temperatures (298.15–1173.15 K) in a dry porous clay sample, in a diffusion chamber. They derived the diffusive transport properties using a pore-scale transport model. Based on the observation of a strong increase in gas diffusivity of CO_2_ compared to O_2_, they suggested that solid–gas interfacial diffusion should be considered to fully understand the diffusion mechanism. The pore-scale transport model was extended to account for interfacial diffusion.

Macroscopic diffusion phenomena are usually described by Fick’s diffusion law.^54^ The first Fick’s law relates the diffusion flux to the concentration gradient, and the second law predicts the influence of diffusion on the time variation of the concentration. The first and second Fick’s laws in one dimension can be written mathematically as follows
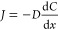
1
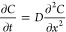
2where *J* is the diffusion flux in mol/m^2^/s, *D* is the diffusion coefficient in m^2^/s, *x* is the position in length (m), *C* is the concentration in amount of substance per unit volume (mol/m^3^) and *t* is the time in seconds (s).

Fick’s law is a widely used fundamental law for the diffusion of gases where the concentration, pressure, and temperature gradients lead to a net mass flux. Fick’s equations are used in most macroscopic numerical simulations and diffusion models.^55−61^ However, Fick’s law has some limitations that make it inapplicable in certain systems.^62^ In addition, Fick’s law is inadequate when dealing with stagnant gases or very slowly diffusing gases, for example, Ar and N_2_.^62^ According to Thorstenson et al.,^62^ an accurate understanding of gas transport in fine-grained porous media must include the possible contribution of Knudsen diffusion, molecular and non-equimolar components of diffusive flux, and viscous (pressure-driven) flux.

Over the years, molecular dynamics simulations have been used to study the transport of fluids in clays.^14,39,63−68^ It is a powerful tool to understand the diffusive mechanism of gas molecules in nanopores as well as the interactions between the fluid of interest and its environment and how these affect the diffusive behavior. These insights are not easily obtained through experimental studies and macroscopic numerical modeling. Apostolopoulou et al.^63^ used molecular dynamics simulations to investigate the transport properties of methane in dry, nanoconfined slit pores as a function of pore width. The methane fluid structure and diffusion coefficient within 5-nm-wide pores were quantified in different pore regions and the results were scaled up using a kinetic Monte Carlo model to predict diffusion in mesopores.^63^ Similarly, Sui et al.^67^ used Grand Canonical Monte Carlo and molecular dynamics methods to study the adsorption and diffusion of shale gas in a dry and wet Na-montmorillonite nanopore at different temperatures and pore sizes. It was found that short-range dispersive interactions affect CH_4_ sorption on clay walls and CH_4_ diffusion increases with increasing pore size.^67^ In addition, it was found that preloading the pore space with water reduces the sorption of CH_4_ in clay, as CH_4_ is hydrophobic and therefore does not mix with water molecules.^67^ Wang et al.^68^ used molecular dynamics simulations to investigate the diffusion behavior of supercritical methane in shale nanopores as a function of the pore size, pore pressure, and moisture content.^68^ One of their findings was that, moisture slows down methane diffusion, but this behavior is more pronounced in organic materials than in inorganic materials, which is due to the clustering and adsorption membrane effect of water in organic and inorganic pores, respectively.^68^ The diffusion coefficient of each gas within a mixture can be significantly influenced by varying compositions of the gases.^69^ Through the use of molecular dynamics simulation, Kadoura et al.^69^ investigated the structural and transport characteristics of CO_2_, CH_4_, and their mixture at a temperature of 298.15 K. The study revealed that the self-diffusion coefficients of CH_4_ experience a notable decrease when exposed to high loadings of CO_2_, primarily due to steric hindrance effects.

Although much progress has been achieved in understanding the diffusion behavior of gas in a clay medium, there are still a number of unresolved issues. The first, concerns the diffusion mechanism of different gas molecules, the influence of system composition, and interactions with the confinement. So far, most studies have focused on the diffusion of CH_4_. The second challenge is that, most studies have focused on either dry clays or fully saturated clays. Studies on partially saturated clays have mostly been conducted at the macroscopic scale and thus,the diffusion of gases at the molecular scale in partial saturation has not been fully understood. The third challenge is that, unlike anions where adsorbed water films become the transport pathway for diffusion in partially saturated systems,^14^ gas transport occurs simultaneously in three domains: (1) diffusion in the gas phase, (2) diffusion in the adsorbed water phase, and (3) diffusion in the transition zone at the gas–water interface.

To address these questions, molecular dynamics is applied here to study the diffusion of molecular gas (He, H_2_, CO_2_, Ar, CH_4_) through partially saturated smectite clay. The influencing factors, e.g., gas type, temperature, and degree of saturation, are analyzed. The partition coefficient, which indicates the retention and retardation capacity of the gas transport, is also analyzed. The simulation is performed under natural conditions at a pressure of 12 MPa, which is typical of the confining pressure expected in the geological repository. Finally, nonequilibrium molecular dynamics (NEMD) is used to study the gas dynamics and hydrodynamics of gas and water molecules confined in partially saturated smectite. In particular, the dynamic viscosity and behavior of the gas–water interface as a function of the degree of saturation are studied. The aim is to gain a comprehensive mechanistic understanding of the diffusion of gases in partially saturated smectite clays, as well as to demonstrate the ability of MD to complement experiments and macroscopic numerical modeling. The obtained results propose a mathematical model required for further investigation in an upscaling approach for the diffusion of gases in clay rocks through multiscale numerical modeling.

## Methods

### System Setup

**Figure 1:**
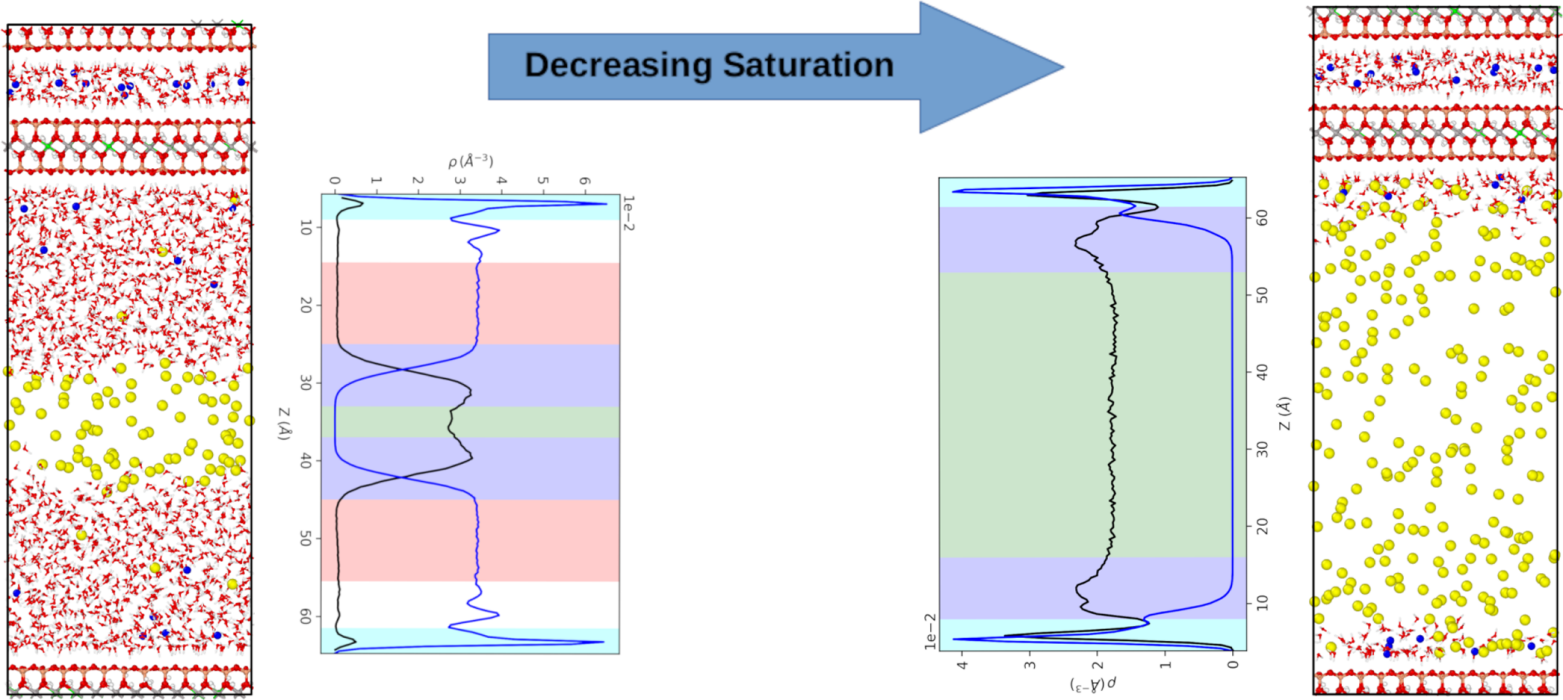
Snapshot from the MD simulation of a 6 nm Na-montmorillonite slit pore for two different degrees of saturation (gas-filled pore width of 1.8 and 5.3 nm; left and right, respectively). Oxygen atoms are red. Hydrogen atoms forming H_2_O molecules and OH groups are white. Silica atoms are pink. Aluminum atoms are gray. Magnesium atoms are green. Sodium atoms are blue. Gas molecules, represented by argon in this particular case are yellow. The graphs next to the snapshots show the density distribution of water and gases in the slit pore. The black curve is the density distribution of the gas molecules (magnified by a factor of 10 for the sake of visualization) and the blue is that of the water. The cyan area on the density plot shows the solid–liquid interface, the pink area shows the domains occupied by the bulk-liquid-like phase, the green area shows the central domain occupied by the gas phase, and the blue area indicates the gas–water interface regions.

Molecular dynamics (MD) simulations were carried out to study the diffusion of different gas molecules in a clay slit pore as a function of temperature and degree of saturation (water to gas ratio in the system). Further analysis was applied to estimate fluid viscosity and gas–liquid partition coefficients. The simulation setup is very similar to the one used by Churakov.^14^ Briefly, the setup consists of a stack of two layers of Na-montmorillonite (tetrahedra–octahedra–tetrahedra sheets) arranged parallel to each other in the *XY* plane with a nanopore of 0.6 nm between them. An external mesopore of about 6 nm was introduced in which the gas-filled pore width as well as the composition of the gas phase could be varied. A snapshot of the simulation cell is shown in Figure 1. The solid phase composition is 2 × 4 Na_0.5_[Al_3.5_Mg_0.5_]Si_8_O_20_(OH)_4_. Isomorphous substitutions of Mg ions for Al ions in the octahedral sheet were randomly distributed to avoid Al–Al pairs in adjacent octahedra. These substitutions create a permanent structural charge on the clay layer, which is compensated by Na ions. A total of 24 isomorphous substitutions were included. To ensure an even distribution of charges in the system, 12 Na ions were placed in the center of the interlayer pore, while 6 Na ions were placed near either side of the mesopore.

### Simulation Details

#### Equilibrium Molecular Dynamics

Molecular dynamics simulations were performed using the open-source package LAMMPS.^70^ The interatomic interaction potentials for clay atoms, water, and gas molecules are the same as those used in our previous work, which also explains the model uncertainties.^42^

The setup and equilibration of the system take full account of the gas molecules partitioning between gas-rich and water-rich phases (solubility) by implementing an iterative equilibration process under imposed PVT constraints. For the sake of data comparison, all of the simulations are conducted in the same d-spacing of 6 nm distance between the TOT layers. The gas-rich phase is confined between the two water-rich films wetting the TOT layers. To achieve the desired density (pressure) and enable gas molecules to dissolve (partition) from the gas phase into the water phase, we use an iterative NPT equilibration process. Initially, the water saturated system was equilibrated in the NPT ensemble at a temperature of 300 K and a pressure of 12 MPa. The final size of the simulation cell after equilibration was 31.17 × 35.88 × 83.72 Å^3^. Then, a fraction of water molecules in the middle of the slit pore was removed from the center of the slit pore and replaced with gas molecules according to a density estimate for the target *P*–*T* conditions (Figure 1). Subsequently, the system was iteratively equilibrated in the NPT ensemble under an external pressure of 12 MPa. This ensemble allows the system to relax and account for the interactions between the gas and the surrounding components. Additionally, it ensures the equilibrium partitioning of gas and water molecules between the water- and gas-rich phases. Since the behavior of a gas under high pressure may deviate from the ideal gas relation, the NPT ensemble is essential for achieving a more realistic representation. To ensure that the system maintain a structure equivalent to the saturated system (with an average pressure of 12 MPa and dimensions of 31.17 × 35.88 × 83.72 Å^3^), the system size and average pressure after equilibration were examined. If these parameters did not match the desired values, the number of gas molecules was adjusted accordingly, and the NPT simulation was repeated. This iterative process was carried out until the target mesopore size of ∼6 nm, and the equilibrium molar density of the gas phase was consistently maintained. The simulations were performed at two temperatures (300 and 330 K) for eight saturation states and five gas species.

For each system, a 10 ns production run with a time step of 1 fs was performed in the NVT ensemble at temperatures of 300 and 330 K after equilibration. The temperature of 330 is close to in situ conditions in an underground repository. The Nosé–Hoover thermostat was applied to keep the temperature constant.^71,72^ A fully flexible CLAYFF force field was applied to the atoms of the clay particles, while the water molecules were kept rigid using the SHAKE algorithm.^73^ Short-range interactions were calculated using the Lennard–Jones potentials with a cutoff of 12 Å. Long-range electrostatic interactions were calculated using the particle–particle particle–mesh method (PPPM) with a cutoff value of 12 Å. The final 8 ns trajectories were split into two blocks to obtain two independent replicates for further uncertainty analysis.

#### Diffusion Coefficient

Due to the coexistence of the two-phase fluid and its strong interaction with the surface of clay minerals, structurally distinct areas were identified in the simulated mesopore (Figure 1). These include (1) the mineral fluid interface with strong density fluctuations, (2) a bulk-liquid-like water film, (3) a transition zone between water-rich and gas-rich regions, and (4) a low-density gas-rich phase. To accurately calculate the diffusion coefficient in the mesopore for gas molecules, the mesopore was divided into three regions: liquid phase, gas-rich phase, and water–gas interface. In this study, we focused only on diffusion in the gas phase and the water–gas interface. Diffusion in the liquid phase was investigated in our previous study on the mobility of gas molecules in saturated smectites.^42^ To obtain the diffusion coefficient in different regions, we adopted the MD-calculated survival probability and the mean square displacement method of Liu et al.^74^ In this study, only the *x* and *y* components of the diffusion tensor, describing the two-dimensional diffusion parallel to the gas–water interface were analyzed. Assuming a layer parallel to the basal surface of the clay particle with intervals in the *z*-dimension defined as {*a*,*b*}, the diffusion coefficient of particles located in the region of the interval {*a*,*b*} in one dimension is given as follows
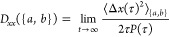
3and
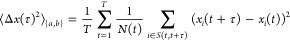
4and
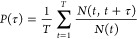
5where ⟨Δ*x*(τ)^2^ ⟩_{*a*,*b*}_ is the mean square displacement of the atoms in the region {*a*,*b*}, *P*(τ) is the survival probability, *T* is the total number of time steps, averaged over, *N*(*t*) denotes the number of particles in the region at time *t*, and *N*(*t*, *t* + τ) is the set of all particles remaining in the region {*a*,*b*} during the time interval between *t* and *t* + τ.

#### Partition Coefficient and Gibbs Free Energy of Molecule Transfer

The equilibrium partitioning of water and gas molecules between coexisting water-rich and gas-rich phases could be determined by molecular dynamics simulations. At equilibrium, the partition coefficient *K* of the gas molecules in the liquid phase is calculated from the ratio of the number densities of gas molecules in the liquid phase (ρ_gas in water phase_) and gas phase (ρ_gas in gas phase_), normalized to the number density of water molecules, (ρ_water in water phase_) and (ρ_water in gas phase_), in each phase, respectively
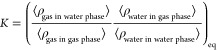
6

From the partition coefficient *K*, the Gibbs free energy difference associated with the transfer of a gas molecule from the gas phase to the liquid phase^75,76^ can be calculated directly as
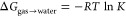
7where *R* and *T* are the molar gas constant (8.3145 J/(mol K)) and the absolute temperature, respectively.

#### Nonequilibrium Molecular Dynamics

To evaluate the effects of viscous stresses in the system and their correlation with diffusion, partial saturation, and gas type, nonequilibrium molecular dynamics was used to study the hydrodynamic properties of the confined fluid. In equilibrium molecular dynamics, the macroscopic impulse of the simulation cell and its angular moment are set and maintained at zero. In nonequilibrium molecular dynamics, a constant external force is applied to the particles in a specific direction. This corresponds to the introduction of a uniform pressure gradient along the given direction in the system, resulting in a flow of particles. In this study, two simulation approaches were used to analyze the dynamics of gas: (a) a constant force acting only on the gas molecules in the gas phase (decoupled gas-phase dynamics (DGPD)) and (b) a constant force acting on all fluid particles (coupled gas and water phase dynamics (CGWPD)). To drive the flow, a constant external force of 5.0 × 10^–3^ kcal/mol in the *x*-direction, parallel to the surface, was applied to each fluid atom. This force is low enough to maintain the linear response conditions between applied stress and fluid velocity. A temperature of 300 K was set with the Nosé–Hoover thermostat and this thermostat was coupled only to the degrees of freedom of the fluid in the *y*-direction, which is along the surface and perpendicular to the fluid flow. The clay particles were kept rigid throughout the simulation. The velocities of the fluid were recorded every 1 ps for 6 ns in bins along the *z*-direction (pore width) with a bin width of 0.1 Å, after a 2 ns equilibration.

At a steady state, the velocity distribution of a pressure-driven flow (*F*_*x*_) in a pore channel can be described by the Poiseuille flow. The Poiseuille flow leads to a parabolic velocity profile that can be described by the Stokes equation for the velocity component in the *x*-direction as a function of the distance from the middle of the slit pore (*h*)
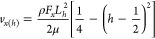
8

The dynamic viscosity of the fluid, assuming constant viscosity and density in the pore, can be computed from the velocity profile as:
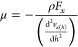
9where *v*_*x*(*h*)_ is velocity along the slit pore width (*h*), *F*_*x*_ is the applied external force, ρ is the number density, μ is the dynamic viscosity, and *L_h_* is the total slit pore length.

## Results and Discussion

### Self-Diffusion of Gas Molecules

The self-diffusion coefficients of gas molecules were first studied by simulating a system of 1000 gas molecules at temperatures of 300 and 330 K and a pressure of 12 MPa for each type of gas. The system was equilibrated in the NVE ensemble for 200 ps. A further equilibration of 1 ns was performed in the NPT ensemble. This was followed by a production run of 5 ns in the NVT ensemble to calculate the self-diffusion coefficients. Table 1 shows the values of the self-diffusion coefficients of gas molecules from the MD predictions.

**Table 1:** Predicted Self-Diffusion Coefficients of Gas Molecules *D* (10^–6^ m^2^/s) at 12 MPa

*T* (K)	H_2_	He	CH_4_	Ar	CO_2_
300	1.470	1.257	0.164	0.134	0.040
330	1.511	1.486	0.217	0.161	0.069

To verify and validate the model parameters and the values of the self-diffusion coefficients, the results of the MD simulations were compared with experimental data^77−80^ and also evaluated using an empirical procedure.^81−83^ Both data sets agree very well with the empirical relationships. Calculations from the kinetic theory of gases have been used in the past to estimate the diffusion coefficient, viscosity, and thermal conductivity of dilute gases using their intermolecular forces.^84^ This approach was extended by Slattery et al.^81^ by developing a relation from dimensional analysis considerations and experimental diffusivities using Enskog’s kinetic theory for dense gases. Their formulation can predict the self-diffusion coefficients of dense nonpolar gases. Based on the work of Slattery and Bird,^81^ Stiel et al.^82^ developed a comprehensive relation for calculating the self-diffusion coefficients of polar, nonpolar, and binary gas mixtures for dense gases.

In this study, the density of gas molecules at equilibrium was used to predict the self-diffusion coefficients using the empirical formulation of Stiel et al.^82^ (eqs 10 and 11) and Slattery et al.^81^ (eq 12). Equations 10 and 11 apply to Ar, H_2_, CO_2_, and CH_4_ gas, while eq 12 applies to He gas
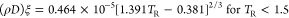
10and
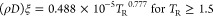
11and
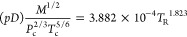
12where ρ is the gas density, *D* is the diffusion coefficient, ξ = *T*_c_^1/6^/*M*^1/2^*P*_c_^2/3^, *p* is the system pressure, *T*_c_ is the critical temperature of gas, *P*_c_ is the critical pressure of gas, *M* is the molecular weight, and *T*_R_ is the reduced temperature (*T*/*T*_c_).

**Figure 2:**
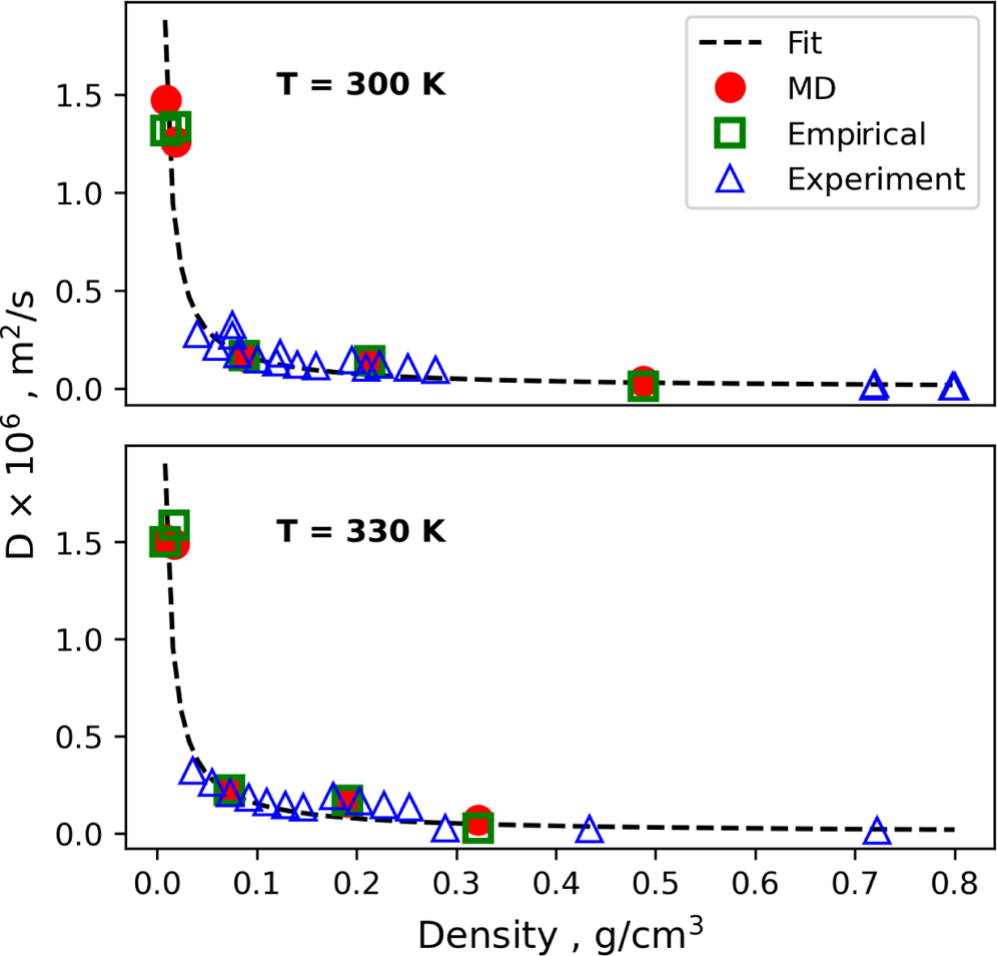
Predicted self-diffusion coefficients for gas molecules as a function of equilibrium gas density at 12 MPa from MD simulations. The graph includes experimental values^77−80^ as well as predictions based on empirical relations. The fitting line shows diffusion coefficients predicted using the Chapman–Enskog theory, described by Chapman and Cowling.^85^

Figure 2 shows a plot of the self-diffusion coefficients of gas molecules as a function of gas density from MD predictions. The graph also shows the values calculated from the empirical procedure and experiments. From Figure 2, it can be seen that the self-diffusion coefficient of gas molecules is inversely proportional to the gas density. The fitting line in Figure 2 represents a fitting of the diffusion coefficients at low densities using the Chapman–Enskog theory described by Chapman and Cowling.^85^

### Gas Partitioning

**Figure 3:**
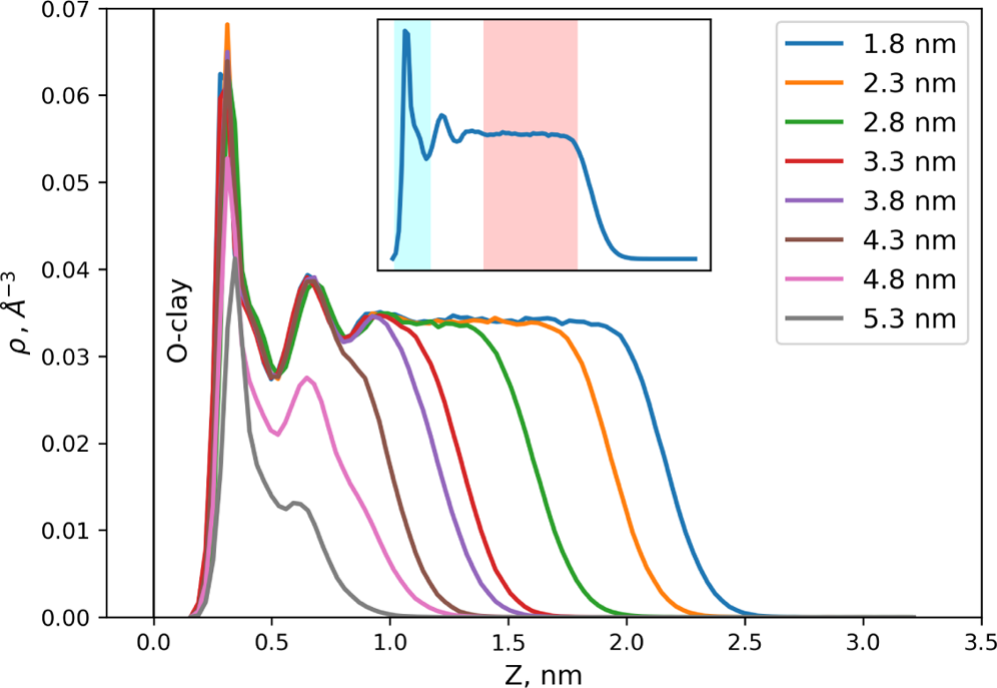
Density distribution of water on the external surface of the clay (half of the pore width). (Inset) The cyan-colored area on the density plot shows the solid interface characterized by strong structuring of fluid. This domain is used for the analysis of gas partitioning to the solid–liquid interface. The pink-colored area shows the domain occupied by the bulk-liquid-like phase.

The distribution of the gas molecules in the water-rich phase was determined from the density profiles (Figures S2–S11) of the gas in the water-rich phase. The partition coefficient (*K*) and the free energy difference (Δ*G*) associated with the transfer of the gas molecules into the water-rich phase are obtained from eqs 6 and 7, respectively. Figure 3 illustrates the density distribution of water on the external surface of the clay as a function of gas-filled pore width. At a smaller gas-filled pore width, the density profile shows three highly structured layers of water at distances of 0.3, 0.6, and 0.9 nm from the surface of the clay. This behavior is typical of what can be expected under fully saturated conditions.^14,42^ Further away from the surface >1.0 nm, the density distribution is constant and the water shows a bulk-like behavior.

Taking into account the structuring of the liquid near the clay surface, the partition coefficients and Gibbs free energy of gas transfer were determined for two regions: (a) the partitioning of gas molecules to the solid–liquid interface and (b) the partitioning to the bulk-like water-rich region. These regions are indicated in Figure 3 by the colors cyan and pink, respectively (inset). The values of gas molecules at the solid–liquid interface (*K*_I_, Δ*G*_I_) and in the bulk-like water region (*K*_B_, Δ*G*_B_) are given in Table 2. For a wide range of saturation, the *K* and Δ*G* values are essentially constant within the statistical uncertainties of the simulation. Therefore, the results in Table 2 represent averaged values over several simulations with different gas-filled pore widths. It is important to note that, in the context of partitioning in the bulk-like water region, our averaging calculations were limited to a range of gas-filled pore widths between 1.8 and 3.3 nm. This restriction was implemented as the region in question is no longer present beyond a gas-filled pore width of 3.3 nm. Similarly, for partitioning at the solid–liquid interface, our results were limited to a range of gas-filled pore widths between 1.8 and 4.3 nm. This was done in accordance with our trajectory analysis, which indicated that for gas-filled pore widths exceeding 4.3 nm (in the case of thin water films), the water molecules tend to cluster around the Na ions, resulting in discontinuous coverage of the clay surface by the water film. As a consequence, gas-filled domains make direct contact with the clay surface.

**Table 2:** Partitioning Coefficients *K* (10^–7^) and Gibbs Free Energy of Transfer *G* (kJ/mol) of Gas Molecules at the Solid–Liquid Interface and at the Bulk-Like Water Region

*T* (K)		H_2_	He	CH_4_	Ar	CO_2_
300	*K*_B_	0.97 ± 0.46	2.45 ± 1.30	1.51 ± 0.82	7.90 ± 1.49	138.97 ± 26.29
	*K*_I_	1.22 ± 0.58	2.56 ± 1.02	1.34 ± 0.66	7.26 ± 2.80	142.14 ± 21.72
	*G*_B_	40.64 ± 1.46	38.29 ± 1.23	39.52 ± 1.24	35.09 ± 0.48	27.94 ± 0.50
	*G*_I_	40.07 ± 1.45	38.06 ± 1.00	39.68 ± 0.99	35.53 ± 1.29	27.87 ± 0.41
330	*K*_B_	10.94 ± 3.95	20.06 ± 4.44	10.05 ± 4.32	53.02 ± 7.21	389.24 ± 58.56
	*K*_I_	9.15 ± 3.80	20.12 ± 3.46	9.83 ± 5.24	49.03 ± 7.46	392.30 ± 78.15
	*G*_B_	37.84 ± 1.00	36.07 ± 0.64	38.16 ± 1.21	33.35 ± 0.36	27.89 ± 0.41
	*G*_I_	38.35 ± 0.97	36.03 ± 0.50	38.44 ± 1.73	33.57 ± 0.39	27.89 ± 0.55

The results indicate that *K*_B_ and *K*_I_ increase as the temperature is increased. Moreover, the particle density of the gas molecules was found to impact gas partitioning. CO_2_ and Ar, which have molecular masses of 44 and 39.95 g/mol, respectively, exhibit significantly higher partitioning coefficients than He, H_2_, and CH_4_. Interestingly, despite He having a smaller molecular mass than CH_4_, its small molecular size allows for easier penetration into the interstitial spaces between water molecules, resulting in greater partitioning. Prior research has demonstrated a direct relationship between partition coefficient and molecular weight,^86,87^ as well as polarizability.^88−91^

### Effect of Saturation on the Diffusion of Gases

**Figure 4:**
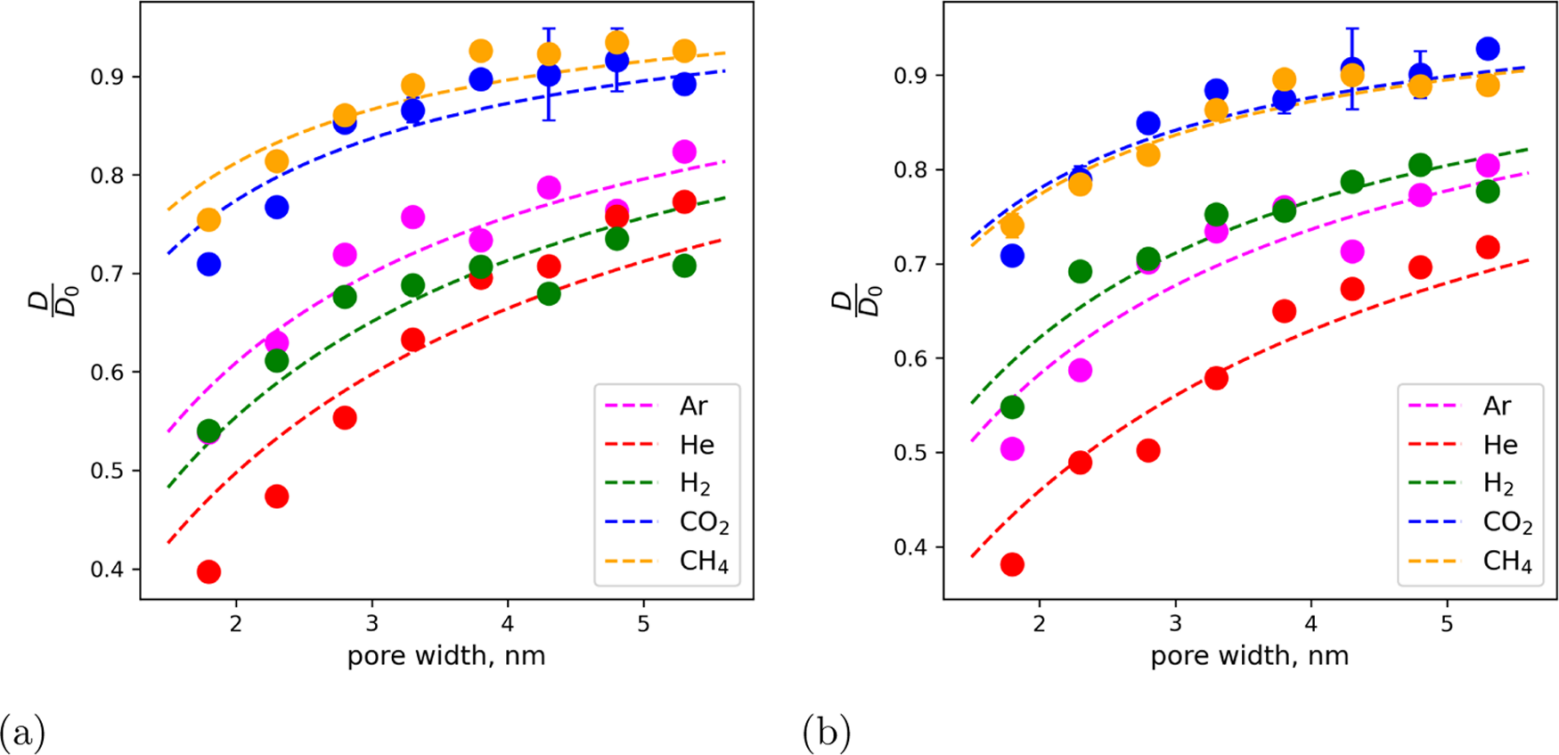
Relative diffusion coefficient of gas molecules in the bulk gas phase in Na-MMT at 12 MPa as a function of gas-filled pore width: (a) 300 K and (b) 330 K.

The self-diffusion coefficient of gas molecules in the gas phase at temperatures of 300 and 330 K as a function of the gas-filled pore width, normalized to the diffusion coefficient in the bulk (*D*_0_), is shown in Figure 4. It should be noted that gas-filled pore widths below 1.8 nm were excluded from this study because a larger quantity of gas molecules was required to keep the pore space open for gas-phase diffusion. This behavior is likely due to dispersive interactions between opposing water platelets that result in a net attractive force between closely spaced platelets. As the gas-phase pore size increases, this effect diminishes.

The results show that the diffusion coefficient increases with the pore size and asymptotically converges toward the value of the bulk state. It was originally assumed that a constant value (*D*_0_) over a wide range of pore widths would be obtained by using the survival probability, which calculates the diffusion coefficient associated exclusively with the gas phase in the bulk, and thus eliminating the effects of confinement. However, the opposite was found. The results showed that He gas specifically was strongly affected by confinement compared to other gases; possibly due to the Knudsen effect. To this effect, the mean-free path of the gas molecules was calculated based on the collisions of gases analysed from the molecular dynamics simulations trajectories. A comparison with the calculations of the mean-free path performed with kinetic theory is made, which agrees well. Table 3 shows the values for the mean-free path from molecular dynamics calculations and kinetic theory for temperatures of 300 and 330 K.

**Table 3:** Mean-Free Path of Gas Molecules (nm) from MD Simulations (λ_MD_) with σ_col_ = σ_gas_ and Kinetic Theory (λ_KT_)^a^

	300 K		330 K	
gas	λ_KT_	λ_MD_	λ_KT_	λ_MD_
H_2_	8.45	10.47	9.13	12.02
He	10.40	10.91	11.65	11.89
Ar	6.23	6.01	6.87	6.31
CH_4_	4.98	4.76	6.09	5.93
CO_2_	3.01	3.04	4.59	5.20

a σ_col_ is the collision diameter and σ_gas_ is the diameter of the gas molecule that is the Lennard–Jones collision diameter (σ).

In general, it can be observed that the maximum gas-filled pore size is still smaller than the mean-free path for most gas molecules. Consequently, the diffusion coefficient is reduced due to collisions with the water surface, which results in gas molecules having a shorter mean-free path under confinement. Strong influence is exerted on He gas because it has the largest mean-free path compared to the other gases.

**Figure 5:**
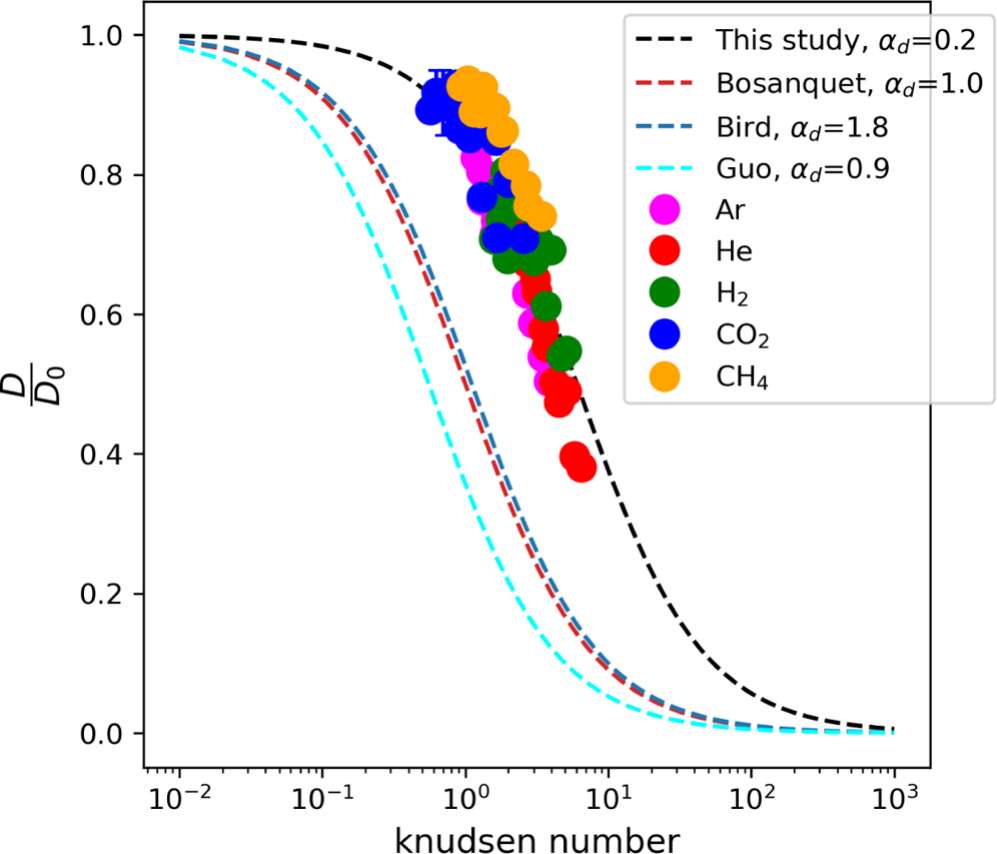
Relative diffusion coefficient of gas molecules normalized to the diffusion coefficient in a pure bulk system as a function of the Knudsen number and fitted with eq 13. The curve shift observed in this study is attributed to the specific physicochemical conditions considered, which differ from the simplified conditions assumed in the original Bosanquet approximation.

The Knudsen number was calculated from the mean-free path and the gas-filled pore sizes. A diagram showing the relationship between the diffusion coefficient and the Knudsen number is shown in Figure 5. A mathematical equation developed from the graph after the Bosanquet approximation^92,93^ is given as
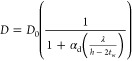
13and
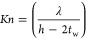
14where *D*_0_ represents the bulk diffusion coefficient in the gas phase at 12 MPa, λ denotes the mean-free path of the gas at 12 MPa, *h* is the width of the pore, *Kn* is the Knudsen number, and *t*_w_ is the thickness of the adsorbed water film, which varies with the degree of saturation. The parameter α_d_ is the Bosanquet correction parameter obtained through fitting and has a value of 0.2.

Based on the work of Yin et al.,^94^ the identification of three gas diffusion regimes can be made in a porous media, determined by the local Knudsen number. The regimes are characterized by *Kn* values of ≤0.1, 0.1 < *Kn* < 10, and *Kn* ≥ 10. In the case of molecular diffusion (*Kn* ≤ 0.1), intermolecular collisions are dominant. When Knudsen diffusion occurs (*Kn* ≥ 10), the dominant collisions are between molecules and a solid wall. In the case of transition diffusion (0.1 < *Kn* < 10), significant intermolecular collisions and collisions between molecules and the solid wall occur. The Knudsen number ranges from 0.6 to 6 in our results, indicating that diffusion is in the transition regime and not purely molecular.

Several studies have shown that the original Bosanquet formula can approximate gas diffusion well in the transition regime.^92,95−97^ Guo et al.^95^ used the Boltzmann transport equation to study gas diffusion and found a Bosanquet correction parameter, α_d_ of 0.9, where the Knudsen number was determined using the method of the largest sphere. The Knudsen diffusivity derived from the kinetic theory of gases yielded an α_d_ of 1.8, which underestimated the value obtained by Guo et al.^95^ In this study, we obtained an α_d_ of 0.2, which appears to overestimate the other predictions. It is worth noting that the original Bosanquet approximation was developed from empirical studies and relies on several idealized conditions. The current system exhibits nonideal gas behavior due to the high pressure. In addition, the gas-wall collisions may not be perfectly elastic since the wall consists of water molecules. Hence, surface phenomena such as adsorption and water vapor mobility at the gas–water interface may not be captured by the original Bosanquet approximation. Chen et al.^98^ investigated the validity of the Bosanquet approximation using equilibrium molecular dynamics simulations and concluded that further modifications to the Bosanquet approximation are necessary to account for the intermolecular forces of fluid molecules and the smoothness of the pore walls.

Experimental data regarding gas diffusion coefficients in partially saturated smectite micropores as a function of gas-filled pore width are currently unavailable. However, a previous investigation of gas diffusion in saturated smectite incorporated a geometric factor and utilized an empirically derived function from a molecular dynamics study.^42^ This approach produced predictions that closely matched experimental gas diffusion data in Boom clay. Therefore, we propose to adopt a similar methodology in our current study and modify eq 13 accordingly
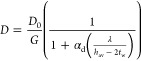
15where *G* is the geometric factor and *h*_av_ is the average pore width. The geometric factor refers to an empirical parameter accounting for the reduction of the diffusive transport of species in porous media in comparison with the bulk phase due to the geometric complexity of pore space.^36^ The geometric factor takes into account the tortuosity of diffusion paths, pore constrictivity, and the overall complexity of the clay pore structure. In general, the tortuosity and constrictivity cannot be measured independently and are thus often lumped in a single parameter referred to as a geometric factor. This equation allows to predict the gas-phase diffusivity for general conditions based on the geometric properties of porous media, mean pore size, and the partial water pressure via the corresponding parameters, *G*, *h*_av_, and *t*_w_. A further effect of temperature can be considered following the approach described in our previous study.^42^

#### Gas–Water Interface Diffusion

Accurately determining the diffusion coefficient at the gas–water interface is challenging due to the imprecise definition of the region encompassing the interface. To address this issue, we have defined the interface region as the area bounded by the adsorbed gas layer at the water interface, indicated by a cyan margin in Figure 1. Figure S1 displays the diffusion coefficient at the gas–water interface. The results indicate that diffusion at the gas–water interface is ∼10% lower than in the bulk. Notably, we observe from Figure S1 that the diffusion coefficient remains constant when the interaction water surface is in the bulk water phase. However, this behavior changes and increases as a function of adsorbed water film thickness for water film thicknesses below 1.0 nm. This is probably due to the structuring of water molecules close to the clay surface.

### Flow Dynamics

The study of fluid behavior necessitates a fundamental understanding of dynamic viscosity. As such, nonequilibrium molecular dynamics simulations were conducted in order to address this objective. A constant force acting on each fluid atom in the direction parallel to the clay surface initiated a fluid flow. Subsequently, steady-state velocity profiles were analyzed to determine the dynamic viscosity of gas in partially saturated Na-MMT. The Navier–Stokes equation (eq 8) predicted a parabolic (Poiseuille) velocity profile assuming constant viscosity and density. Using eq 9, the dynamic viscosity was then obtained. These findings provide valuable insights into the dynamic properties of fluids in confined spaces.

#### Method Validation

**Figure 6:**
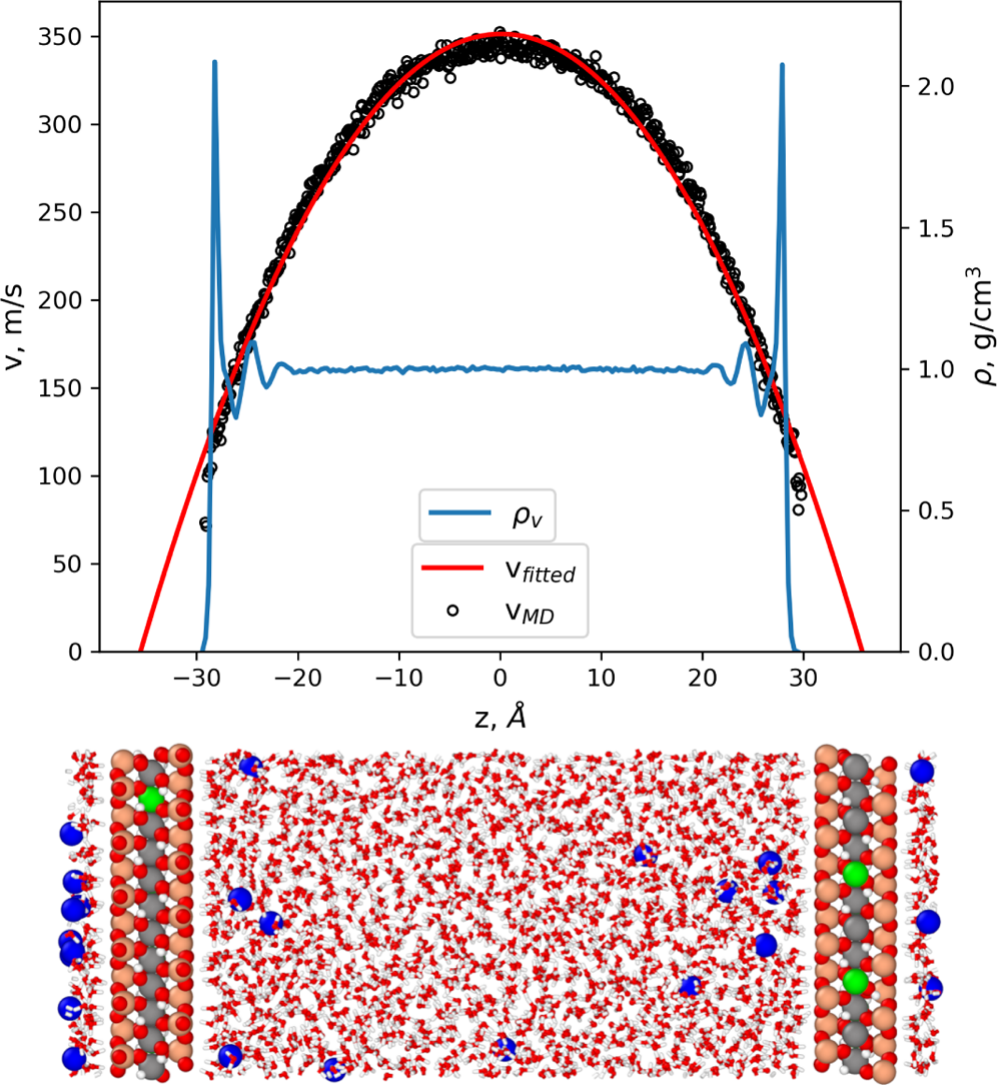
Steady-state velocity profile of water flow in Na-MMT. The force is acting parallel to the surface on each fluid atom in the *x*-direction. This diagram also shows the mass density profile of water. The black markers represent the velocity values from the MD simulation, the red line is the parabolic fit of the Poiseuille flow extrapolated here to the velocity at zero, and the blue line represents the mass density profile of water.

To validate the simulation setup, nonequilibrium molecular dynamics simulations were conducted for water flow in fully saturated Na-MMT. The resulting velocity and density profiles are shown in Figure 6. The density profile of water at the center of the pore remains constant at 1 g/cm^3^, indicative of the bulk water behavior. Close to the surface, notable fluctuations in water density are observed within a 1 nm region, with a maximum density of ∼2 g/cm^3^. The calculated dynamic viscosity for water in a 6 nm nanopore is 0.634 cP, This value that is in agreement with prior studies on the dynamic viscosity of bulk water,^99^ flow simulations in a 6 nm Na-MMT nanopore,^99^ and experiments with bulk water,^100^ which reported values of 0.66, 0.68, and 0.891 cP, respectively. In contrast to the findings of Boţan et al.,^99^ deviations from the parabolic flow profile near the surface were not significant in this study. This may be due to the use of a constant force, which is ∼1 magnitude of order higher than that utilized in their study.^99^ A higher force leads to greater water mobility, thereby diminishing the impact of water–solid surface interactions. The sliding velocity at the surface was roughly 75 m/s, indicating higher mobility than the study by Boţan et al.,^99^ which reported a velocity of about 4 m/s.^99^

The boundary condition responsible for the parabolic profile at the center of the pore is a crucial parameter that has been extensively studied.^101^ The slip boundary condition has conventionally been assumed for continuum-scale fluids. In their review on microfluidics, Squires et al.^101^ demonstrated that the no-slip boundary condition is not valid for gas within distances smaller than the mean-free path from the wall. They also showed that noncontinuum effects play a role in the boundary conditions of fluids confined to a molecular scale. Experimental techniques used to study microfluidic behavior generally indicate that wetting (hydrophilic) surfaces follow the no-slip boundary condition, whereas nonwetting (hydrophobic) surfaces follow a slip boundary condition.^101^ To reveal the appropriate boundary conditions best applicable to the system and estimate the slip at the solid–liquid and liquid–gas interface, the slip length was calculated for each interface. The slip length is defined as the distance within the surface/wall at which the (extrapolated) fluid velocity would be stationary; this can be expressed as
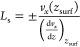
16where *L*_s_ is the slip length, *v*_*x*_ is the slip velocity at the solid-liquid or liquid–gas boundary, *z*_surf_ is the location of the slip surface, and  is the derivative of the slip velocity at the slip surface.

The simulation resulted in a slip length value of 5.00 ± 0.45 Å for flow of water in Na-MMT. Although there are no experimental determinations of the slip length, this value is in agreement with the value of 6 Å obtained by Marry et al.,^102^ when studying electroosmosis in Na-MMT.

#### Gas Dynamics in Slit Pore

As an initial approach, the bulk viscosity of gas species was determined through bulk equilibrium molecular dynamics simulations, utilizing the Green–Kubo method at a temperature of 300 K and pressure of 12 MPa. The obtained results, presented in Table 4, indicate good agreement with the experimental values obtained at a temperature of 300 K and pressure of 0.1 MPa. It is thereby demonstrated that the dependence of viscosity on pressure is moderate. Nevertheless, the molecular weight presents a strong influence on the viscosity values.

**Table 4:** Dynamic Viscosity (Centipoise) and Slip Length (Å) Values for Gas Dynamics

gas	μ_0_	μ_exp_^a^	μ_dry_	Div (%)	*L*_DGPD_	*L*_CGWPD_	*L*_dry_
H_2_	0.009	0.009	0.007	–24.4	3.94 ± 1.02	4.29 ± 0.52	41.78
He	0.018	0.020	0.013	–29.2	3.16 ± 0.34	4.24 ± 0.67	54.14
Ar	0.028	0.023	0.021	–1.3	1.24 ± 0.32	1.81 ± 0.35	2.98
CH_4_	0.010	0.011	0.004	–5.0	0.65 ± 0.19	0.94 ± 0.45	8.58
CO_2_	0.033	0.015	0.014	83.3	0.74 ± 0.13	2.06 ± 0.47	0.65

a μ_exp_ is experimental dynamic viscosity values.^103−107^ μ_0_ denotes the viscosity of gas from the bulk gas system using the Green–Kubo method. μ_dry_ is the viscosity of gas in a dry pyrophyllite. *L*_DGPD_ denotes the slip length for gas in the DGPD. *L*_CGWPD_ denotes the slip length for gas in a CGWPD. *L*_dry_ denotes the slip length for gas in the dry pyrophyllite. The symbol Div represents the percent difference between the relative viscosities in the maximum partially saturated gas-filled pore width (μ/μ_0_) and in the dry pore width (μ_dry_ /μ_0_) .

**Figure 7:**
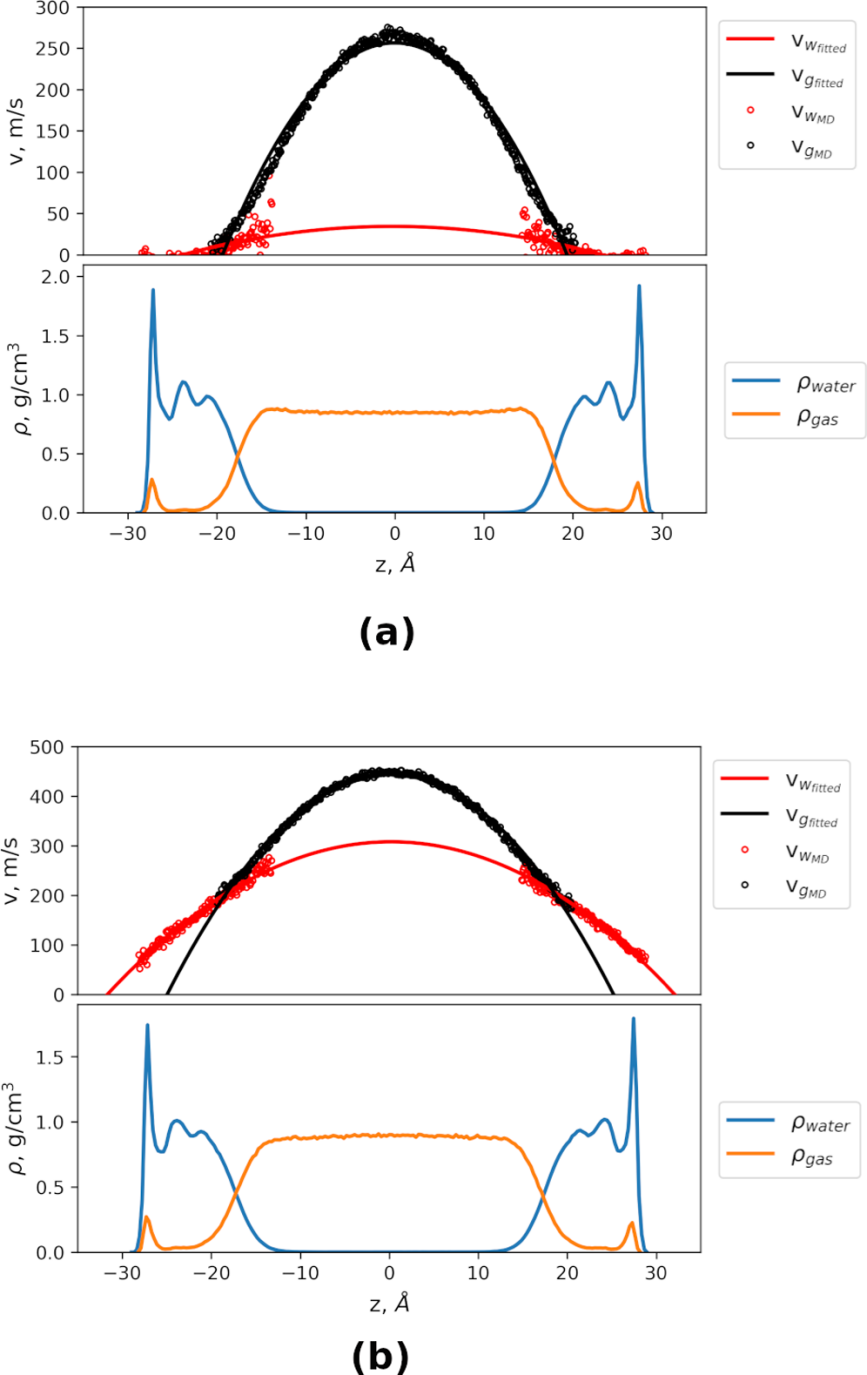
Velocity and density profiles of CO_2_ gas in steady state at a pore width of 3.3 nm. The red line fits the velocity profile of water, the black line fits the velocity profile of CO_2_ gas, the blue is the density profile of water and the orange is the density profile of CO_2_ gas. (a) DGPD and (b) CGWPD.

As previously noted, two distinct methodologies have been developed for investigating the dynamics of gas species, as depicted in Figure 7: decoupled gas-phase dynamics (DGPD) and coupled gas and water phase dynamics (CGWPD). In the DGPD system (Figure 7a), the adsorbed water film experiences no external forces but rather undergoes weak traction at the gas–water interface due to the coupling of shear stresses resulting from the flow of gas species.

**Figure 8:**
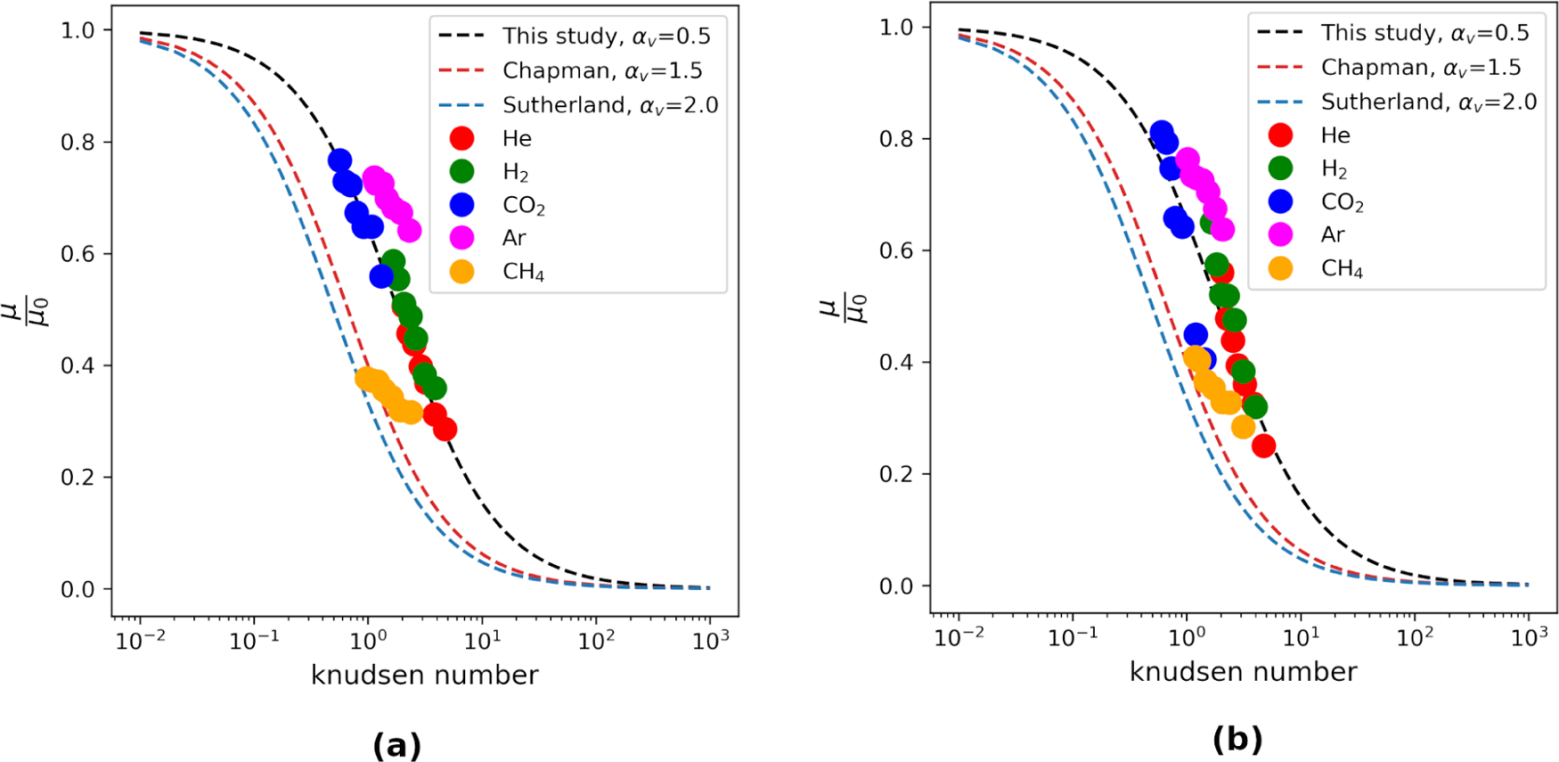
Dynamic viscosity of gas molecules in Na-MMT as a function of the Knudsen number: (a) DGPD and (b) CGWPD.

The dynamic viscosity of the gas species is shown in Figure 8 as a function of the Knudsen number for DGPD and CGWPD. The findings demonstrate that the viscosity behavior remains unchanged in both DGPD and CGWPD, indicating that dynamic viscosity is an inherent property of the gas. However, a strong dependence of viscosity on the Knudsen number is observed. This means that the pore size and mean-free path of a gas under confinement are crucial in determining the viscous behavior of the gas. Again, a Bosanquet-type approximation that satisfactorily describes the Knudsen number dependence of the viscosity is given (with an introduction of the geometric factor for macroscopic predictions)
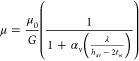
17where μ_0_ represents the bulk dynamic viscosity of the gas, λ denotes the mean-free path of the gas, *h*_av_ is the average width of the pore, *t*_w_ is the thickness of the adsorbed water film, which varies with the degree of saturation, and α_d_ is the Bosanquet correction parameter obtained through fitting and has a value of 0.5. Here, *G* is a geometric factor, which is a function of the permeability.

Based on the Knudsen number, four flow regimes are recognized by a widely used empirical classification of gas flow in rarefied conditions.^108^ The Navier–Stokes equation is the best tool to describe flow when *Kn* < 10^–3^ because, the continuum assumption is valid and is supported by the no-slip boundary condition at the walls. With slip-velocity and temperature-jump boundary conditions, the continuum equations’ applicability can be expanded for the regime of 10^–3^ < *Kn* < 10^–1^ (also known as the slip-flow regime).^109,110^ The transition regime 10^–1^ < *Kn* < 10^1^ is the most challenging to handle. Intermolecular collisions for *Kn* > 10^1^ become negligible, and the kinetic theory is typically used to describe the so-called free-molecular flow regime.^111^

This study investigates the transition regime of the viscous flow of gas species. The rarefaction (correction) parameter α_v_ for this study is found to be 0.5, which differs from the values reported in previous studies, such as 1.5 and 2.0.^111^ This deviation is attributed to the same underlying factor as that discussed for diffusion. To explore the impact of the pore wall type on viscosity, gas flow simulations were conducted in a dry pyrophyllite. The viscosity values and the corresponding percentage deviation of the relative viscosities in the maximum partially saturated gas-filled pore width (μ/μ_0_) and in are presented in Table 4. The observed deviation values (Div) of the gas species indicate a pronounced influence of the interacting surface on the dynamic viscosity of the confined gas molecules. Specifically, He, H_2_, CH_4_, and Ar exhibit a higher affinity toward the liquid wall compared to the solid wall, resulting in reduced relative viscosities upon contact with water molecules. He and H_2_ with lower molecular weights show a much stronger affinity. Conversely, CO_2_ exhibits the opposite trend, with a very strong affinity for the clay solid wall.

Table 4 presents the slip length values (*L*_DGPD_, *L*_CGWPD_, and *L*_dry_) for gas flow in DGPD, CGWPD, and dry pyrophyllite. The results demonstrate that both DGPD and CGWPD exhibit a marginal slip condition at the liquid surface, with CGDW exhibiting slightly higher slip length values than DGPD. This observation may be attributed to the presence of water molecules moving tangentially to the gas flow at the gas–water interface, resulting in dynamic stresses exerted on the gas surface due to relative motion, which leads to additional traction. The gas flow characteristics of dry pyrophyllite exhibit a more pronounced slip condition compared to those in contact with water, which is an intriguing finding. The slip length is notably larger for helium (He) and hydrogen (H_2_), and this trend correlates well with the divergence (Div) values. He and H_2_ have less affinity for the solid surface, leading to their greater mobility at the interface, resulting in higher slip velocity and subsequently increased slip length. Conversely, CO_2_ exhibits a strong affinity for the solid surface, resulting in a considerably lower slip length.

### Viscosity of Water in Two-Phase Flow

**Figure 9:**
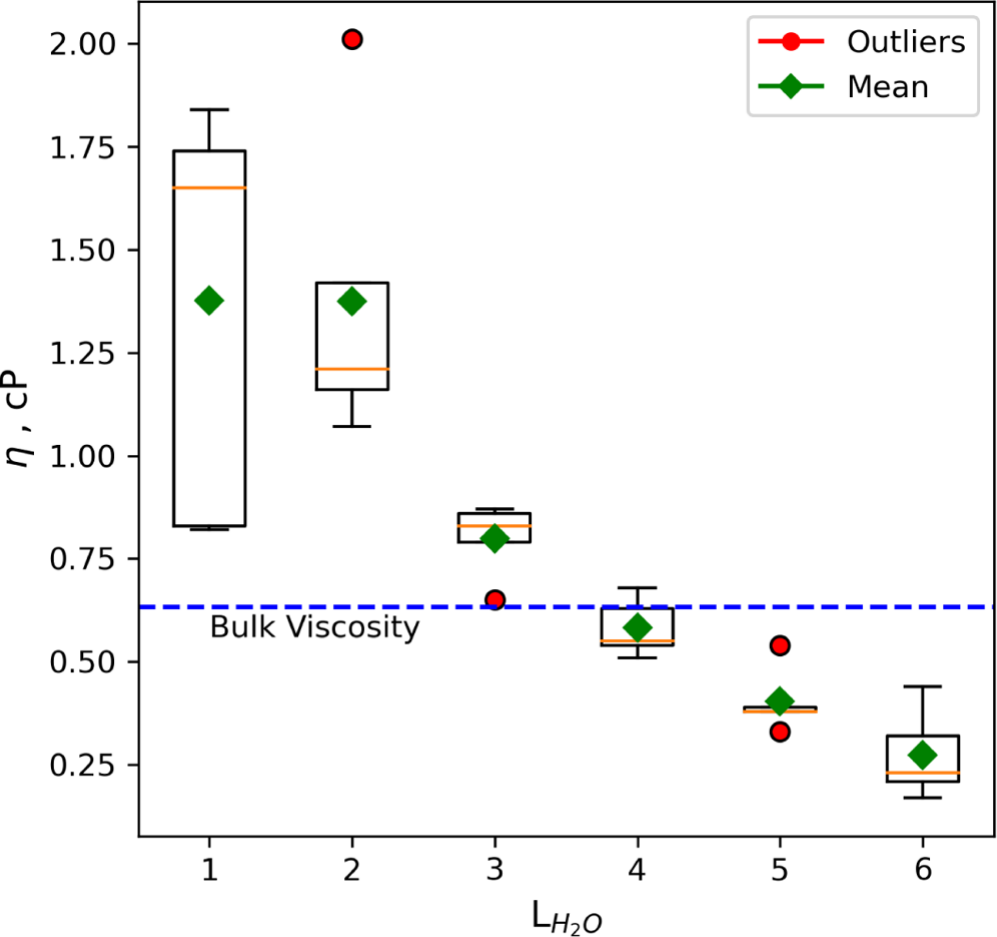
Dynamic viscosity of water films confined in the Na-MMT clay slit pore as a function of the number of adsorbed water layers.

The present study investigates the dynamic viscosity of the water film under partially saturated conditions for CGWPD. Figure 9 displays the dynamic viscosity as a function of the number of adsorbed water layers, which indicates the thickness of the adsorbed water film. The findings reveal that the dynamic viscosity of water increases with decreasing thickness of the adsorbed water film. This is consistent with the hypothesis that water molecules near the clay surface have lower mobility, which leads to a higher dynamic viscosity due to the interaction with the clay surface. At 3.5–1.8 water layers, the dynamic viscosity increases to about 2.0 cP, which is approximately twice the value observed in bulk water. This increase in viscosity is attributed to the fact that, in thin water films, water is more influenced by the clay surface, and mobility is lowered. These findings provide insights into the impact of adsorbed water thickness on water phase mobility near the clay surface and their effect on the dynamic viscosity of water in a CGWPD.

## Conclusions

In this study, a comprehensive understanding of the mobility of gaseous molecules in partially saturated smectite clay (Na-montmorillonite) is provided using molecular dynamics simulations. The porosity of the fluid is subdivided into four distinct domains, including the solid–liquid interface, bulk-like liquid phase, liquid-gas interface, and bulk-like gas phase, based on density profiles. The diffusion coefficients of five types of gaseous molecules (Ar, He, H_2_, CO_2_, and CH_4_) are separately studied for diffusion in the gas phase and at the gas–water interface. It is found that diffusion in the gas phase increases with the gas-filled pore width, asymptotically converging to the diffusion of gas in the bulk state. Furthermore, the diffusion coefficient at the gas–water interface is observed to remain constant as long as it is confined by well-defined bulk-like liquid and gas domains. The system’s diffusive behavior is in a transition regime that comprises a mixture of molecular diffusion and Knudsen diffusion, thus rendering Fick’s law unsuitable for describing the process. To overcome this challenge, a Bosanquet-type approximation is used to describe the effective diffusion coefficient as a function of the adsorbed water film thickness, gas mean-free path, geometric factor, and average pore width, yielding highly satisfactory results.

Furthermore, an investigation into the partitioning of gases in the water-rich phase was conducted. Based on density profiles for gas molecules in two water-rich regions of interest in the system: (a) solid–liquid interface and (b) bulk-liquid-like phase, the partition coefficient and Gibbs free energy of gas transfer were determined. At the solid–liquid interface, adsorbed water layers are formed due to the interaction between water molecules and the clay surface. Our findings demonstrate that the partition coefficient is highly dependent on the temperature and molecular weight of the gaseous molecules.

The intrinsic nature of viscosity is confirmed by nonequilibrium molecular dynamics simulations at different degrees of saturation, where the dynamic viscosity of gases in decoupled gas-phase dynamics (DGPD) and combined gas and water phase dynamics (CGWPD) is found to be identical. Our modeling further reveals that the no-slip boundary condition often used in continuum models is unsuitable for strongly confined fluids and that the finite slip boundary condition should be applied instead. Moreover, the dynamic viscosity of water in CGWPD is observed to increase with decreasing thickness of the absorbed water film. Furthermore, our simulations demonstrate that the dynamic viscosity of gases is highly influenced by the interacting wall surface. These parameters are crucial input parameters for the subsequent step in this study, which involves the development of an upscale pore-scale model.

## Supporting Information Available

Plot of the gas diffusion coefficient as a function of gas-filled pore width at the fluid–gas interface; density distribution diagrams for gases and water; and velocity profile of gas flow in the gas-filled pore (PDF)
