# Supplementary material for: Diffusion and Gas Flow Dynamics in Partially Saturated Smectites
Source: J Phys Chem C Nanomater Interfaces. 2023 Jul 17;127(29):14425–38. doi: 10.1021/acs.jpcc.3c02264 (PMC10389780; doi:10.1021/acs.jpcc.3c02264)
Supplement: Supplementary file 1 — jp3c02264_si_001.pdf [file jp3c02264_si_001.pdf]

Diffusion and Gas Flow Dynamics in Partially Saturated Smectites

Jerry P. Owusu,[*,†,‡] Konstantinos Karalis,[‡] Nikolaos I. Prasianakis,[†] and Sergey V. Churakov[*,†]

†*Laboratory for Waste Management, Paul Scherrer Institute, 5232 Villigen-PSI, Switzerland*

‡*University of Bern, Institute of Geological Sciences, 3012 Bern, Switzerland*

E-mail: jerry-peprah.owusu.psi.ch; sergey.churakov@psi.ch

Surface Diffusion

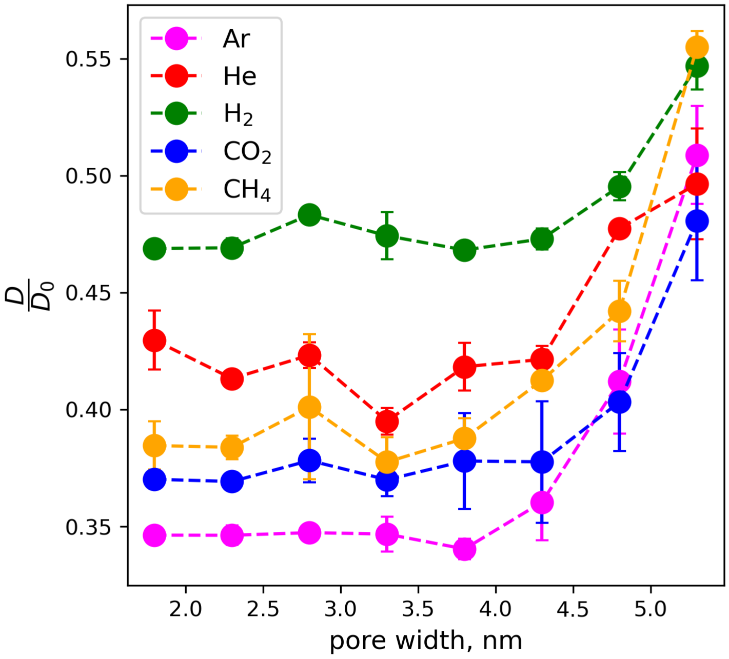

(a)

(b)

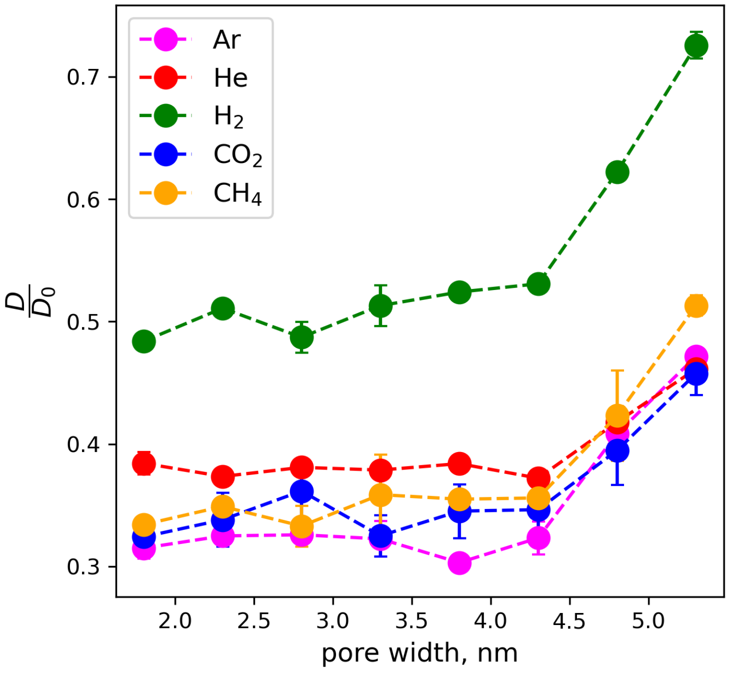

Figure S1: Diffusion coefficient of gas molecules in the gas-water interface in Na-MMT at 12 MPa as a function of gas-filled pore width (a) 300 K (b) 330 K.

Density profiles of gaseous molecules and water

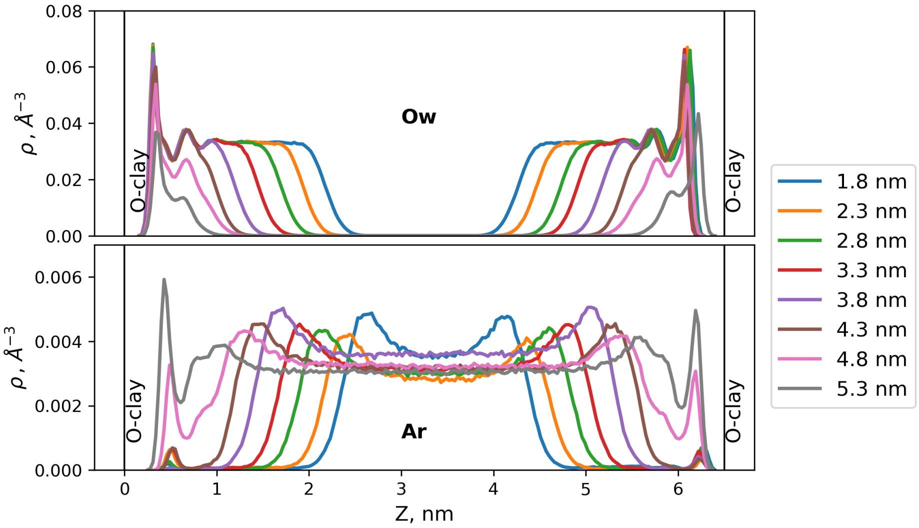

Figure S2: Ar at 300 K

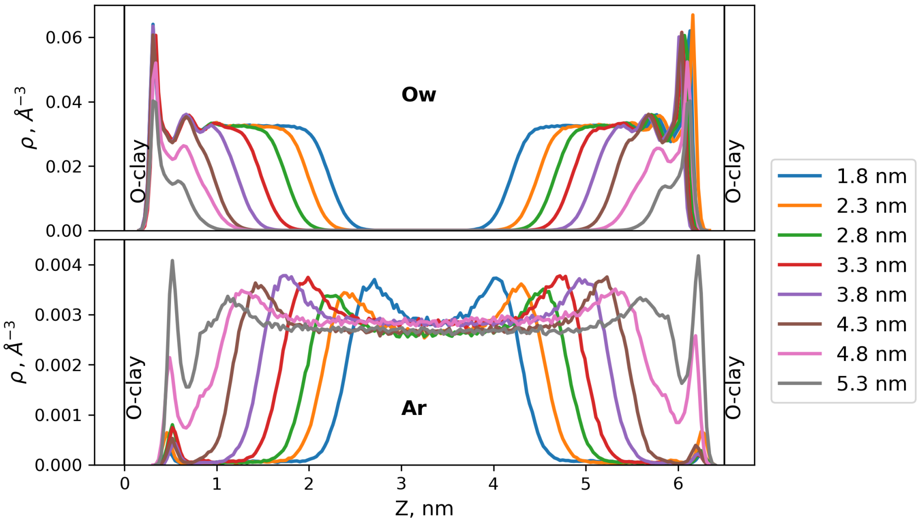

Figure S3: Ar at 330 K

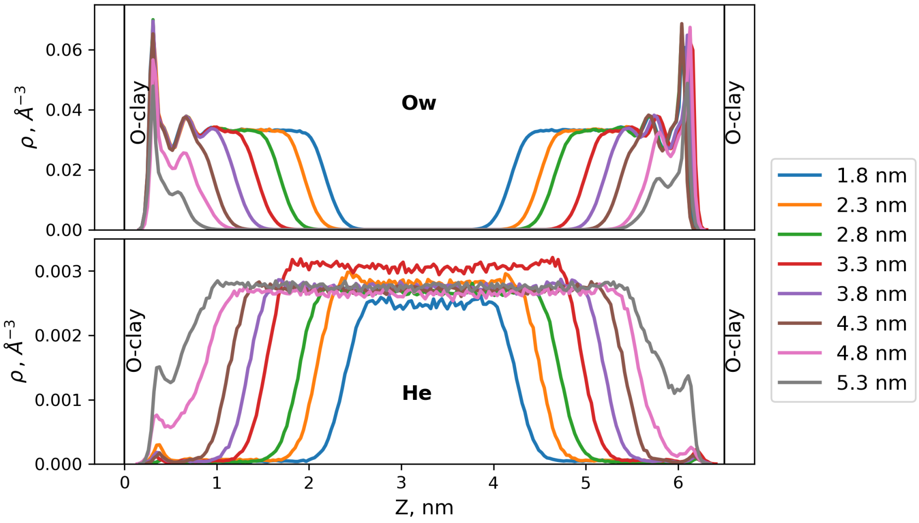

Figure S4: He at 300 K

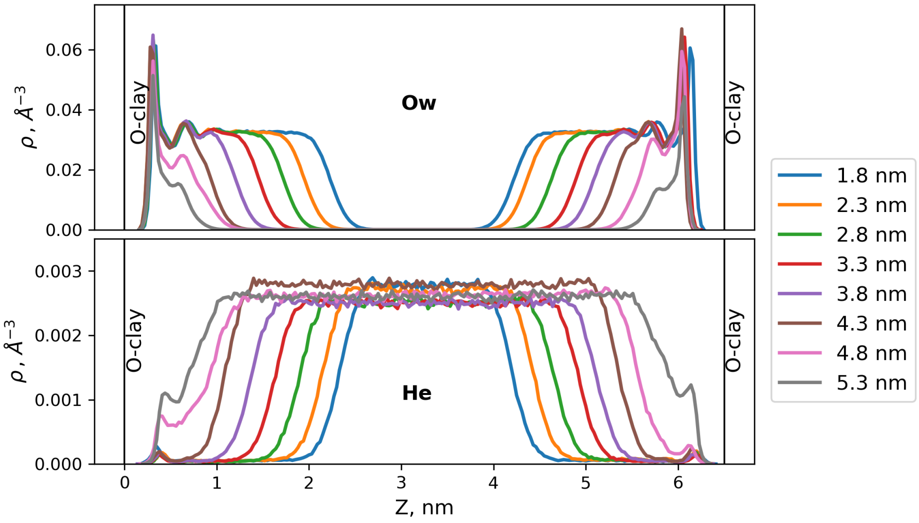

Figure S5: He at 330 K

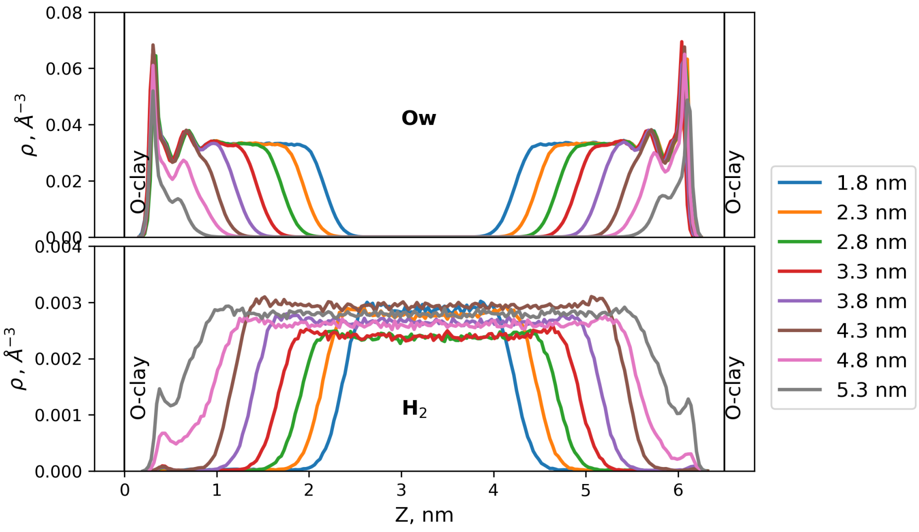

Figure S6: H_2 at 300 K

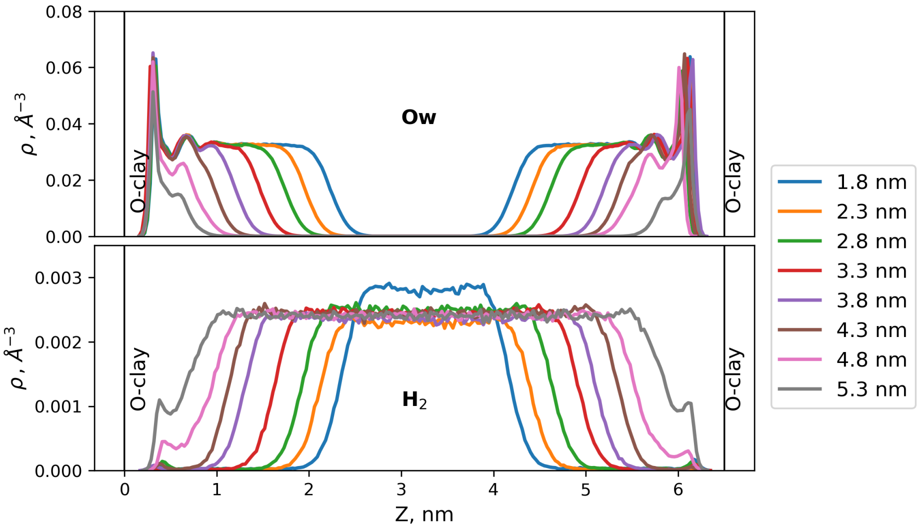

Figure S7: H_2 at 330 K

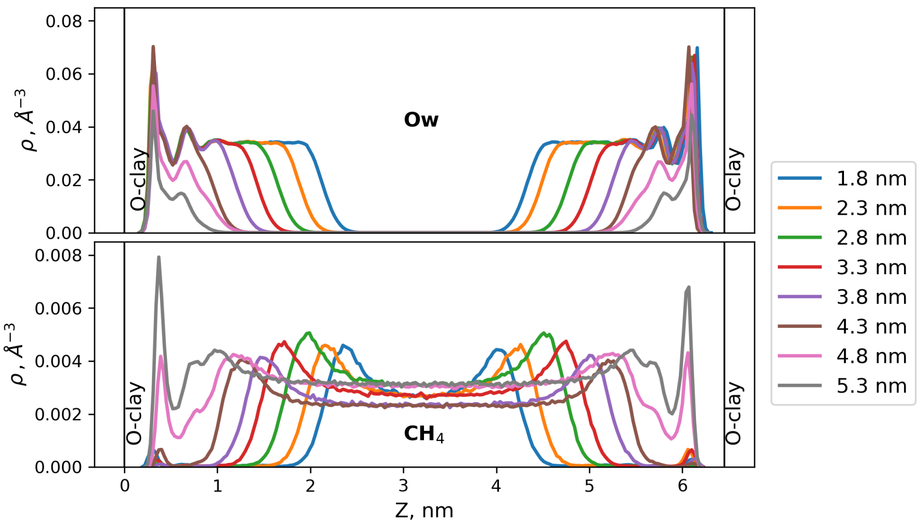

Figure S8: CH_4 at 300 K

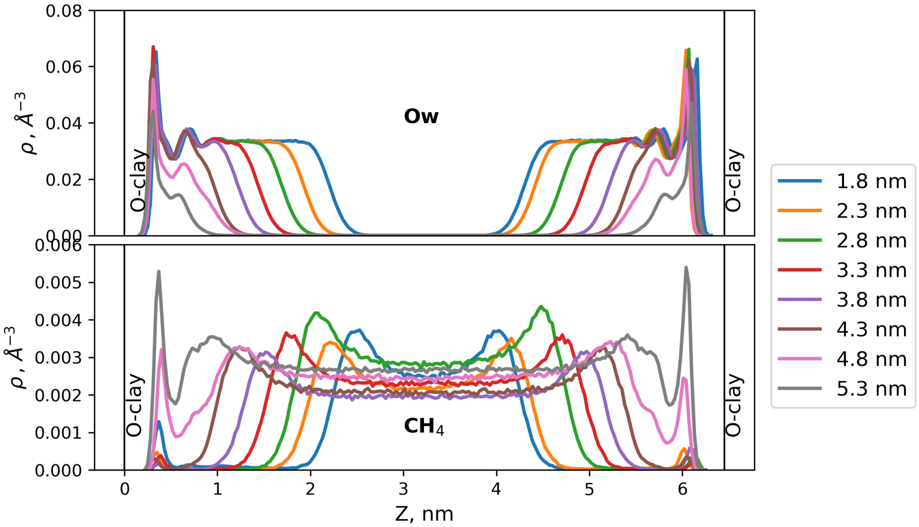

Figure S9: CH_4 at 330 K

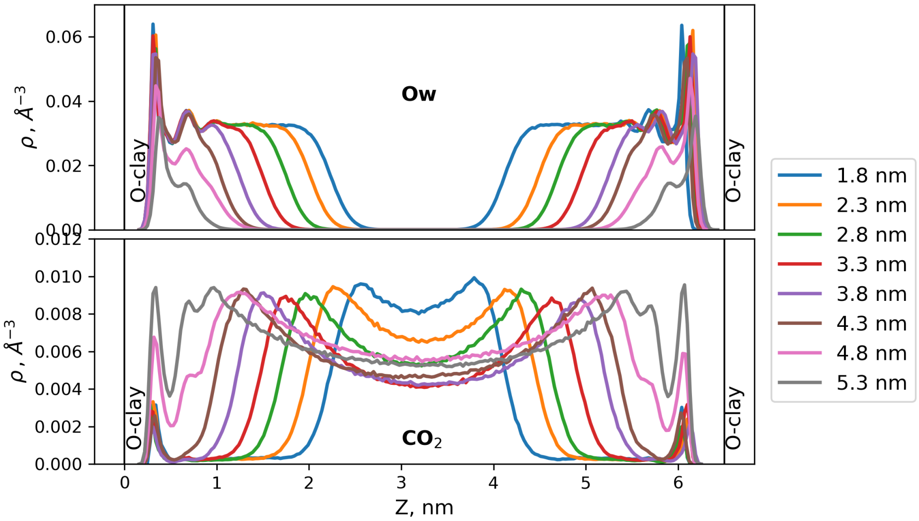

Figure S10: CO_2 at 300 K

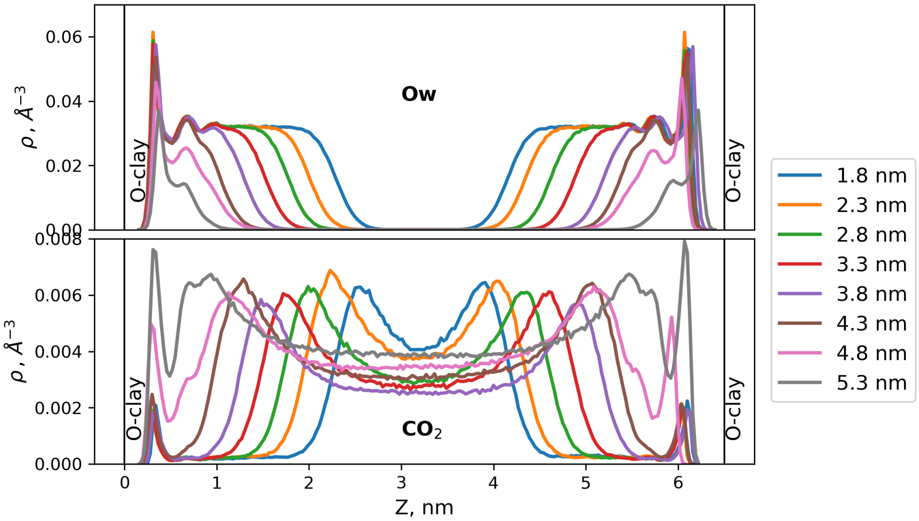

Figure S11: CO_2 at 330 K

velocity profiles of gas flow in slit pore

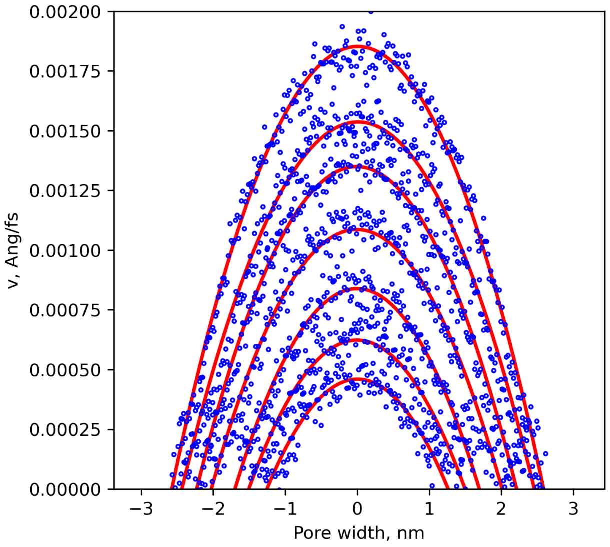

Figure S12: Velocity profiles of Ar

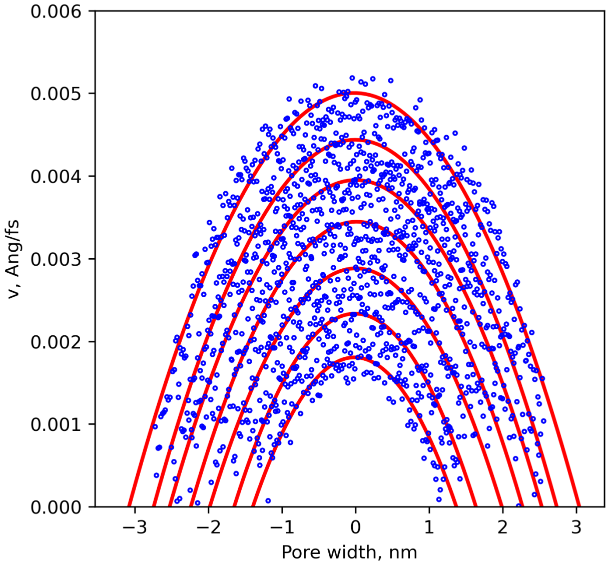

Figure S13: Velocity profiles of He

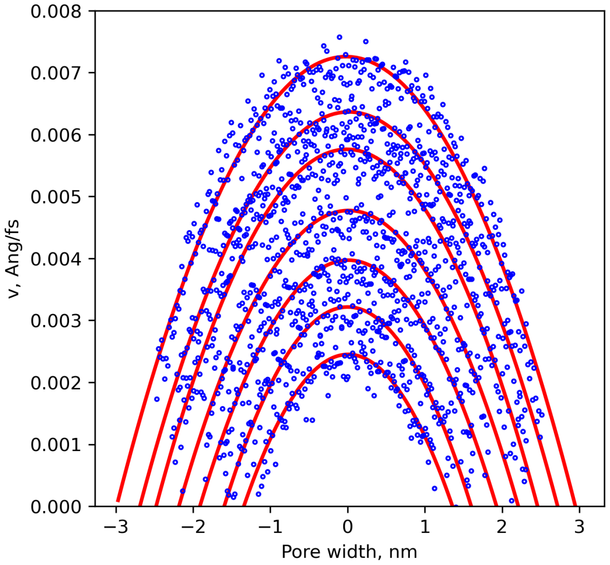

Figure S14: Velocity profiles of H_2

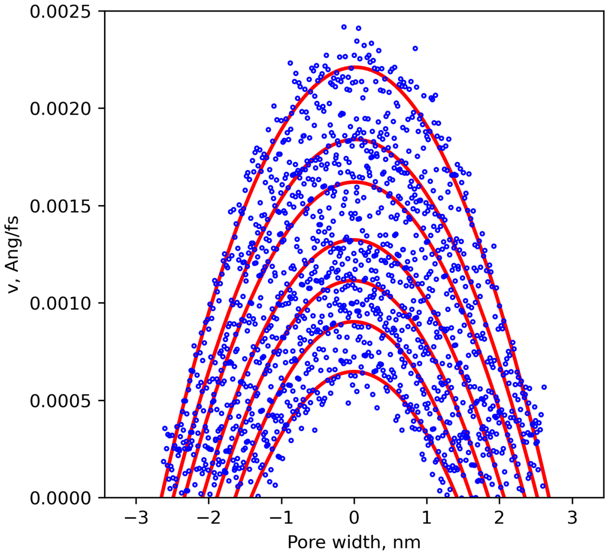

Figure S15: Velocity profiles of CH_4

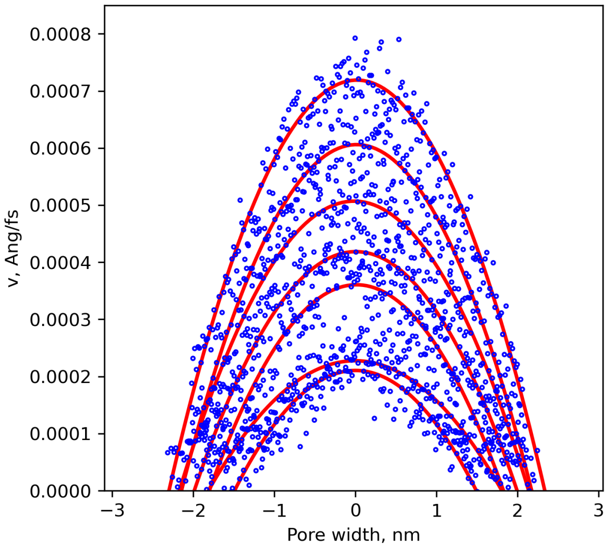

Figure S16: Velocity profiles of CO_4
